# Left heart remodelling in hypertensive patients: a comprehensive echocardiography and computed tomography study

**DOI:** 10.3389/fcvm.2023.1295537

**Published:** 2023-11-24

**Authors:** Aleksandra Lange, Viktoria Palka, Chris Bian, Harry Huntress, Jill Morgan, Sean Allwood, Rohan Swann, Przemysław Palka

**Affiliations:** ^1^ Queensland Cardiovascular Group, Brisbane, QLD, Australia; ^2^ Faculty of Humanities, University of Queensland, Brisbane, QLD, Australia

**Keywords:** hypertension, left atrial function, left ventricular hypertrophy, left heart, echocardiography, diastasis, left ventricular-to-left atrial ratio, computed tomography

## Abstract

**Objectives:**

This study aimed to assess left heart remodelling changes in hypertension, excluding underlying ischaemic heart disease, utilising computed tomography coronary angiography (CTCA) and transthoracic echocardiography (TTE).

**Methods:**

A total of 178 patients (mean age 60 ± 9 years, 53% female) were enrolled in the study: Group 1 consisted of patients with essential hypertension (*n* = 96, Group 1), and Group 2 served as age-matched controls (*n* = 82, Group 2). All participants underwent both CTCA and TTE. TTE measurements included left ventricle (LV) concentricity and function and left atrial (LA) volume and function. Using both CTCA and TTE, we measured LV diastasis volume (LV_dias_) and LA diastasis volume (LA_dias_).

**Results:**

LV mass index and LV mass/height^2.7^ were similar in both the groups. However, Group 1 had a higher prevalence of concentric LV remodelling, characterised by a larger mean LV wall thickness, increased relative wall thickness ratio, and a reduced ratio of LV end-diastolic volume (LV_ED_) index to mean wall thickness (55 ± 14 vs. 65 ± 15, *p* = 0.0007). Group 1 showed higher LA_dias_ and LA minimal volumes, while LA reservoir function was lower in Group 2. The LV_dias_/LA_dias_ ratio was lower in Group 1 compared to Group 2 (TTE 1.77 ± 0.61 vs. 2.24 ± 1.24, *p* = 0.0025, CTCA 1.50 ± 0.23 vs. 1.69 ± 0.41, *p* = 0.0002). A composite score based on four combined TTE parameters, namely, LV_ED_ index/mean wall thickness ≤57, ratio of early diastolic mitral inflow to mitral annular tissue velocities (E/e’) >8, LV_dias_/LA_dias_ ≤1.62, and LA reservoir function ≤0.58, yielded the highest discriminatory power (area under the curve—AUC = 0.772) for distinguishing patients with hypertensive heart disease (HHD). Collectively, we refer to these parameters as the LEDA score, with each parameter scored as one point. For LEDA scores of 0, 1, 2, 3, 4, the probability of underlying HHD was 0%, 23%, 59%, 80%, and 95%, respectively. Furthermore, a CTCA-derived LV_dias_/LA_dias_ ≤1.76, considered as a single parameter, demonstrated modest accuracy in differentiating patients with HHD (AUC = 0.646).

**Conclusions:**

The TTE LEDA score, based on four parameters, namely, LV_ED_ index/mean wall thickness, E/e’, LV_dias_/LA_dias_, and LA reservoir function, proved to be the most effective in defining left heart remodelling in hypertension.

## Graphical Abstract

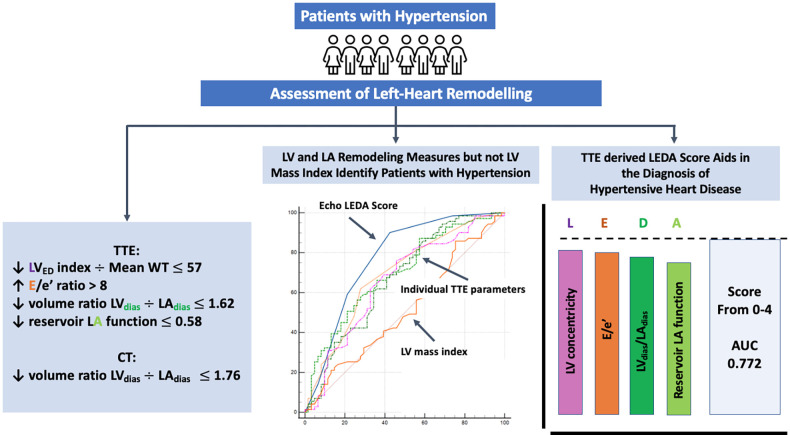

## Introduction

While there have been significant global improvements in the treatment and control of hypertension, there remains a critical need for more timely detection of this condition. This is because the cumulative effects of hypertension can ultimately lead to cardiac overload, remodelling, and heart failure (HF) (1–3). It is noteworthy that each month of active antihypertensive therapy has been associated with a one-day prolongation of life expectancy without cardiovascular death (4). Despite the well-established link between hypertension and increased all-cause mortality, the challenge lies in detecting early adaptive stages of cardiac remodelling (5–9). At present, current transthoracic echocardiogram (TTE) criteria primarily focus on assessing the effects of the maladaptive processes that occur at a stage when the left ventricular (LV) wall thickness and mass (10–13) are already increased, and LV filling has progressed to an advanced or irreversible stage, accompanied by dilatation of both the left atrium (LA) and LV (5, 8). However, it has been shown that the most favourable prognosis for patients with hypertension occurs during the adaptive stage (14, 15). Therefore, it has been proposed that an easily obtainable TTE score for the early detection of cardiac remodelling could significantly aid in treatment and improve patient prognosis. To date, there has been limited investigation into the impact of hypertension in the absence of underlying ischaemic heart disease on left heart remodelling using a combination of two-dimensional and Doppler TTE data. Our study seeks to address this gap in knowledge and assess the clinical significance of left heart remodelling indices, including LV concentricity, filling pressure, LA function, and the LV/LA volume ratio, with particular focus on the concept of atrioventricular interplay (14, 15). Moreover, our investigation includes the assessment of the diastasis LV/LA volume ratio, which we measured using both TTE and computed tomography coronary angiography (CTCA). This ratio serves as a surrogate for global (net) left heart atrioventricular adaptive remodelling in patients with hypertension. By elucidating the effects of hypertension on left heart remodelling in its early stages, we aim to contribute valuable insights that could inform more proactive clinical approaches and ultimately improve patient outcomes.

## Methods

**Figure 1:**
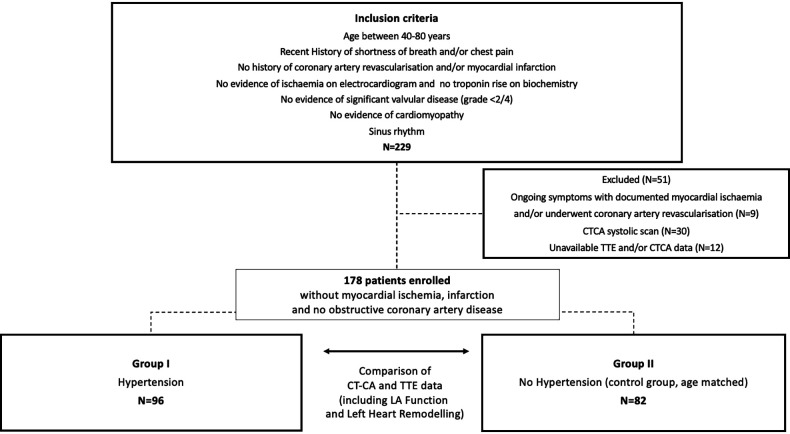
Study design.

This study included patients aged 40–80 years who were referred for CTCA between February 2019 and August 2021. Patients with shortness of breath and/or chest pain were selected from our clinical consecutive referral cohort if they met the following criteria: (1) sinus rhythm, (2) absence of valvopathy ≥2/4 and/or cardiomyopathy, and (3) CTCA and TTE of diagnostic image quality. Patients with documented myocardial ischaemia and/or history of myocardial infarction or coronary artery revascularisation were excluded. The initial group consisted of 229 patients, with 51 patients excluded for the following reasons: nine of 51 patients were excluded because of subsequently documented myocardial ischaemia requiring coronary artery revascularisation. A total of 30 of the 51 patients were excluded as their CTCA was a systolic scan. A total of 12 of the 51 patients were excluded because of incomplete TTE or CTCA data (Figure 1). The final study group comprised 178 patients, divided into two groups: Group 1 consists of patients with diagnosed hypertension (*n* = 96) and Group 2 contains an age-matched control group that included patients who had no clinical evidence of hypertension or diabetes (*n* = 82). Hypertension was diagnosed according to the European Society of Cardiology guidelines (13).

Data were prospectively collected, analysed, and made available upon reasonable request to the corresponding author. This study complied with the principles of the Declaration of Helsinki. It was approved by the Uniting Care Health Human Research Ethics Committee (number 2019.29.307) of Brisbane, Australia. Informed consent was obtained from all patients.

TTE was performed using standard equipment and protocols (Philips EPIQ CVx, Philips North America Corporation, Andover, MA) or Siemens SC200 (Siemens Medical Solutions, Mountain View, CA, USA). A standard clinical imaging protocol was used for each patient. The protocol consisted of M-mode, two-dimensional, and Doppler analyses (16, 17). Apical views were optimised to avoid foreshortening of the LV and/or LA. LV volume was measured using the biplane Simpson method at end-diastole (LV_ED_), end-systole, and diastasis (LV_dias_). The LA area was planimetered from the four-chamber and two-chamber views, excluding the LA appendage and pulmonary veins. LA volume was calculated using the biplane area-length method. LA volume was measured in three phases of the cardiac cycle: maximum (LA_max_) at LV end-systole, diastasis (LA_dias_), and minimum (LA_min_) at LV end-diastole. LA function parameters included (i) LA reservoir function, total emptying volume, and emptying fraction; (ii) LA conduit function, LA passive emptying volume, and emptying fraction; and (iii) LA pump function, LA active emptying volume, and emptying fraction, as previously described (18). Each measurement was averaged over the three cardiac cycles. All the patients were divided according to the recommendations of the American Society of Echocardiography/European Association of Cardiovascular Imaging for the classification of LV diastolic dysfunction (17).

CTCA was performed in a standard manner using a 256iCT scanner (Philips North America Corporation, Andover, MA, USA). The iCT had a 270-ms gantry rotation time and temporal resolution of 135 ms. The collimation scan parameters were 128 mm × 0.625 mm. An intravenous injection of iodinated contrast media (Omnipaque 350) was used to opacify the coronary arteries and, subsequently, the LA and LV. The scan was acquired during a single breath hold, using the prospective electrocardiogram-gated axial step-and-shoot/sequential technique. Only patients scanned in diastasis between 75% and 81% of the R-R interval were chosen for the analysis, with the standard acquisition occurring at 78%. The LA volume was measured by excluding the LA appendage. The LV volume was measured using an automated algorithm within the cardiac software of the Philips Intellispace Portal. The diastolic function assessment, that is, the diastolic expansion index, was measured as the ratio between diastasis LV and LA volume (LV_dias_/LA_dias_) (19).

### Statistical analysis

Continuous variables are expressed as mean ± standard deviation, and categorical variables are expressed as numbers and percentages. The two-sample *t*-test, *χ*^2^ test, Fisher exact test, or non-parametric Wilcoxon/Kruskal–Wallis rank-sum test was used when appropriate for comparison between Group 1 and Group 2. Pearson's correlation coefficient was used to estimate the degree of association between two quantitative variables.

For predefined TTE and CTCA measurements, logistic regression analysis with the DeLong method was used to assess the area under the receiver operating characteristic curve and Youden index J point with a cut-off value to differentiate patients from Groups 1 and 2. Sensitivity and specificity were calculated in a standard manner. Multivariate analysis of variance (MANOVA) was performed to determine the difference in complicated and uncomplicated subgroups of hypertension compared with the age-matched group. A multivariate correlation coefficient or likelihood ratio was used to determine the relationship between echo score and clinical parameters. For all analyses, *p*-values <0.05 were considered significant. All analyses were performed using JMP 15 (SAS Institute, Cary, NC, USA) and MedCalc Version 20.109 (Oostende, Belgium).

## Results

Patient characteristics are presented in Table 1.

**Table 1:** Patient characteristics.

Parameter, mean ± standard deviation or number (%)	Group 1 hypertension (*N* = 96)	Group 2 control (*N* = 82)	*p*-value
Age (years)	61 ± 7	59 ± 10	0.1069
Female gender	44 (56)	40 (49)	0.2564
Body surface area, m^2^	1.96 ± 0.24	1.92 ± 0.23	0.2331
Body mass index	29 ± 6	27 ± 6	0.0327
Office blood pressure systolic, mmHg	139 ± 15	123 ± 9	<0.0001
Office blood pressure diastolic, mmHg	80 ± 8	74 ± 7	<0.0001
Blood pressure (grade)			
Optimal to high normal	46 (48)	82 (100)	
Grade 1	35 (36)	—	
Grade 2	14 (15)	—	
Grade 3	1 (1)	—	
Heart rate, beats/min	67 ± 11	65 ± 13	0.1725
Smoking history (all ex-smoker)	11 (11)	11 (13)	0.6930
Obstructive sleep apnoea	37 (39)	27 (33)	0.4359
Hyperlipidaemia	68 (71)	41 (50)	0.0044
Type II diabetes mellitus	14 (15)	0	<0.0001
Chronic kidney disease, Stage 3	8 (18)	0	<0.0001
Depression anxiety stress score^a^	4.8 ± 3.6	4.5 ± 4.2	0.6067
Quality of life (EuroQol 5 Dimension 5 Level) score^b^	77 ± 18	77 ± 19	0.8483
Blood pressure medications (number)			
0–1	53 (55)	—	—
2	23 (24)	—	—
≥3	20 (21)	—	—
Coronary artery disease			
Grade 1–2 (mild to moderate plaque)	25 (26)	11 (13)	0.0408
Grade 3	0	0	**—**

^a^ DASS-21 (20).

^b^ EQ-50–5l (21).

Group 1 had a higher body mass index, higher rates of hyperlipidaemia, Stage 3 chronic kidney disease, and often mild-to-moderate coronary artery plaques. Complicated hypertension with diabetes or Stage 3 chronic kidney disease was observed in 28 (29%) patients. The depression anxiety stress score (20) and quality of life score (21) were similar in both the groups.

### Comparison of two-dimensional echocardiographic data

As shown in Table 2: Patients in Group 1 had increased mean LV wall thickness (mm) (0.90 ± 0.16 vs. 0.83 ± 0.14, *p* = 0.0042) with no differences in LV diameter, LV_ED_, or LV_dias_. The ratios of LV_ED_ to mean wall thickness and LV_ED_ index to mean wall thickness were lower in Group 1 than in Group 2 (106 ± 29 vs. 119 ± 31, *p* = 0.0061; and 55 ± 14 vs. 62 ± 15, *p* = 0.0007, respectively). The LV relative wall thickness ratio was higher in Group 1 (0.39 ± 0.09 vs. 0.35 ± 0.08, *p* = 0.0029). Concentric LV remodelling was more frequent in Group 1 than in Group 2 [28 (29%) vs. 10 (12%), *p* = 0.0062].

**Table 2:** Echocardiographic (A) and computed tomography data (B).

Parameter	Group 1 (Hypertension)	Group 2 (Control)	*p*-value
(A)			
Mean LV wall thickness, cm	0.90 ± 0.16	0.83 ± 0.14	0.0042
LV diameter, cm	4.7 ± 0.6	4.8 ± 0.7	0.2264
LV diameter/height, cm/m	2.7 ± 0.3	2.8 ± 0.7	0.3568
LV diameter/height, cm/m (>3.3 Women, >3.4 Men)	0 (0)	2 (2)	0.2108
LV mass index, g/m^2^	73 ± 20	70 ± 18	0.3690
LV mass/height^2.7^, g/m	33 ± 9	31 ± 9	0.1178
LV_ED_, ml	94 ± 25	98 ± 26	0.3037
LV_ED_ index, ml/m^2^	48 ± 10	51 ± 10	0.0667
LV_dias_, ml	77 ± 21	80 ± 24	0.3473
LV_dias_ index, ml/m^2^	39 ± 9	41 ± 10	0.0883
LV ejection fraction, %	62 ± 5	62 ± 5	0.7830
LA_max_, ml	66 ± 26	61 ± 24	0.1532
LA_max_ index, ml/m^2^	34 ± 12	31 ± 11	0.1964
LA_max_ index >34, ml/m^2^	43 (45)	30 (37)	0.2877
LA_max_/height^2^, ml/m^2^	22 ± 8	20 ± 7	0.0663
LA_max_/height^2^, ml/m^2^ (>16.5 Women, >18.5 Men)	68 (71)	45 (55)	0.8606
LA_dias_, ml	48 ± 20	41 ± 17	0.0194
LA_dias_ index, ml/m^2^	24 ± 9	22 ± 8	0.0348
LA_min_, ml	31 ± 14	26 ± 14	0.0204
LA_min_ index, ml/m^2^	16 ± 7	14 ± 7	0.0333
E/A, ratio	1.1 ± 0.4	1.2 ± 0.5	0.0960
e’ septal, cm/s	7 ± 2	8 ± 2	0.0233
e’ later, cm/s	8 ± 2	10 ± 3	0.0022
E/e’ (average), ratio	10 ± 3	8 ± 3	0.0006
E/e’>14, cm/s	6 (6)	2 (2)	0.2879
Estimated right ventricular systolic pressure, mmHg	25 ± 10	23 ± 9	0.2595
Tricuspid regurgitation, >2.8 m/s	12 (12)	3 (4)	0.0537
s’ right ventricular, cm/s	12 ± 3	12 ± 2	0.8528
Atrial reversal-A-wave (duration), ms	15 ± 46	13 ± 33	0.7606
Diastolic LV dysfunction grade^a^			
Normal	54 (56)	57 (70)	0.0877
Abnormal	24 (25)	5 (6)	0.0009
Indeterminate	18 (19)	20 (24)	0.3667
LV concentricity and LV hypertrophy			
Relative wall thickness, ratio	0.39 ± 0.09	0.35 ± 0.08	0.0029
LV concentric remodelling^b^	28 (29)	10 (12)	0.0062
LV_ED_/mean wall thickness, ratio	106 ± 29	119 ± 31	0.0061
LV_ED_ index/mean wall thickness, ratio	55 ± 14	62 ± 15	0.0007
LV mass/body surface area, g/m^2^ (>95 Women, >115 Men)	4 (4)	1 (1)	0.3758
LV mass/height, g/m^2.7^ (>47 Women, >50 Men)	3 (3)	3 (3)	1.0000
LV to LA diastolic coupling index			
LV_dias_/LA_dias_, ratio	1.77 ± 0.61	2.24 ± 1.24	0.0025
LA function parameters^c^			
Reservoir (global) function			
Total emptying volume, ml	35 ± 19	34 ± 18	0.8827
Total emptying fraction, %	51 ± 17	56 ± 15	0.0288
Conduit function			
Passive emptying volume, ml	22 ± 15	23 ± 15	0.6431
Passive emptying fraction, %	32 ± 17	36 ± 16	0.0688
Pump function			
Active emptying volume, ml	13 ± 11	11 ± 8	0.3249
Active emptying fraction, %	26 ± 24	30 ± 20	0.2443
(B)			
LV mass index, g/m^2^	65 ± 13	64 ± 14	0.4549
LV_dias_, ml	129 ± 31	135 ± 33	0.2636
LV_dias_ index, ml/m^2^	66 ± 12	70 ± 14	0.0699
LA_dias_, ml	87 ± 20	82 ± 20	0.0722
LA_dias_, index, ml/m^2^	44 ± 9	43 ± 9	0.1576
LV_dias_/LA_dias_, ratio	1.50 ± 0.23	1.69 ± 0.41	0.0002

^a^ As per Nagueh et al. (17).

^b^ Relative wall thickness ratio >0.42 and LV mass index female ≤95 or male ≤115 g/m^2^.

^c^ As per Blume et al. (18).

There were no differences between the groups for the following measurements: LV mass index, LV mass/height^2.7^, and LV ejection fraction.

The analysis of the LA showed that in Group 1, the LA was bigger for the following measurements: LA_dias_ (ml) (48 ± 20 vs. 41 ± 17, *p* = 0.0194), LA_dias_ index (ml/m^2^) (24 ± 9 vs. 22 ± 8, *p* = 0.0348), LA_min_ (ml) (31 ± 14 vs. 26 ± 14, *p* = 0.0204), and LA_min_ index (ml/m^2^) (16 ± 7 vs. 14 ± 7, *p* = 0.0333). LA_max_ as the absolute value or indexed (by either body surface area or height^2.7^) did not differ between Groups 1 and 2. The LV_dias_/LA_dias_ (ml) was lower in Group 1 compared to Group 2 (1.77 ± 0.61 vs. 2.24 ± 1.24, *p* = 0.0025). Among all the studied LA function parameters, only the LA reservoir function measured by LA total emptying fraction (%) was lower in Group 1 compared to Group 2 (51 ± 0.17 vs. 56 ± 15, *p* = 0.0288).

#### Comparison of Doppler data

Table 2: Group 1 had lower e’ values (cm/s) for both the septum (7 ± 2 vs. 8 ± 2, *p* = 0.0233) and lateral wall (8 ± 2 vs. 10 ± 3, *p* = 0.0022). The mean value of the ratio of early diastolic mitral inflow to mitral annular tissue velocities (E/e’) averaged for both the septum and lateral wall was higher in Group 1 (10 ± 3 vs. 8 ± 3, *p* = 0.0006).

#### Comparison of combined criteria for LV diastolic dysfunction

Table 2, LV diastolic dysfunction was more common in Group 1 than in Group 2 [24 (25%) vs. 5 (6%), *p* = 0.0009]. However, a similar percentage of patients in Groups 1 and 2 had normal or indeterminate diastolic function.

### CT data

Table 2: As noted in the TTE data, there was no difference between the groups in terms of the LV mass and LV mass index. There was no difference in LV_dias_ or LA_dias_ between the groups, whether considering the absolute value or the indexed body surface area. The ratio of LV_dias_/LA_dias_ was lower in Group 1 than in Group 2 (1.50 ± 0.23 vs. 1.69 ± 0.41, *p* = 0.0002, respectively).

There was a good correlation between TTE LV_dias_/LA_dias_ and CT LV_dias_/LA_dias_ [for the whole group: *r *= 0.559, confidence interval (CI) 0.448–0.652, *p* < 0.0001; for Group 1: *r *= 0.431, CI, 0.252–0.581, *p* < 0.0001; for Group 2: *r *= 0.560, CI, 0.391–0.693, *p* < 0.0001].

Both TTE and CTCA parameters were analysed using receiver operating characteristics to identify patients with hypertensive heart disease (HHD), see Table 3. We followed the original concept of classifying symptoms into two categories: disease and no disease. Predictive modelling for CTCA data showed that the LV_dias_/LA_dias_ ratio ≤1.76 had discriminatory power to differentiate patients with HHD with an area under the curve (AUC) of 0.646 (*p *= 0.0006). The LV_dias_/LA_dias_ ratio ≤ of 1.76 had a high sensitivity of 91% but low specificity of 36%.

**Table 3:** Cut-off values and power analysis of (A) echocardiographic- and (B) computed tomography–derived parameters for diagnosis of hypertensive heart disease.

Variable	Cut-off value	Area under the receiver operating characteristic curve	95% confidence interval	Youden index J	Sensitivity %	Specificity %	Z-statistics	*p*-value
(A)								
Mean LV wall thickness, cm	≥0.80	0.615	0.540–0.687	0.197	77	42	2.743	0.0061
LA_dias_, ml	>40	0.593	0.517–0.666	0.226	63	60	2.179	0.0293
LA_dias_ index, ml/m^2^	≥20	0.588	0.512–0.662	0.193	66	54	2.070	0.0384
LA_min_, ml	>21	0.619	0.543–0.690	0.210	77	44	2.804	0.0050
LA_min_ index, ml/m^2^	≥12	0.608	0.532–0.680	0.210	70	51	2.536	0.0112
e’ septal, cm/s	≤8	0.611	0.522–0.695	0.290	81	48	2.188	0.0287
e’ lateral, cm/s	≤10	0.668	0.571–0.756	0.378	79	59	3.195	0.0017
E/e’ (average)	>8	0.690	0.604–0.768	0.332	61	72	4.144	<0.0001
LV remodelling/concentricity								
LV relative wall thickness, ratio	>0.40	0.622	0.546–0.693	0.225	40	83	2.917	0.0035
LV_ED_/mean wall thickness, ratio	≤124	0.630	0.555–0.701	0.209	76	45	3.097	0.0020
LV_ED_ index/mean wall thickness, ratio	≤57	0.651	0.576–0.721	0.255	62	63	3.660	0.0003
LV to LA diastolic coupling								
LV_dias_/LA_dias_	≤1.62	0.619	0.543–0.690	0.190	53	60	2.793	0.0052
LA function								
LA reservoir function^a^	≤0.58	0.600	0.524–0.672	0.186	70	49	2.337	0.0194
(B)								
LV_dias_/LA_dias_, ratio	≤1.76	0.646	0.571–0.716	0.272	91	36	3.437	0.0006

^a^ Left atrial total emptying fraction [(LA_max_ − LA_min_)/LA_max_] (18).

**Figure 2:**
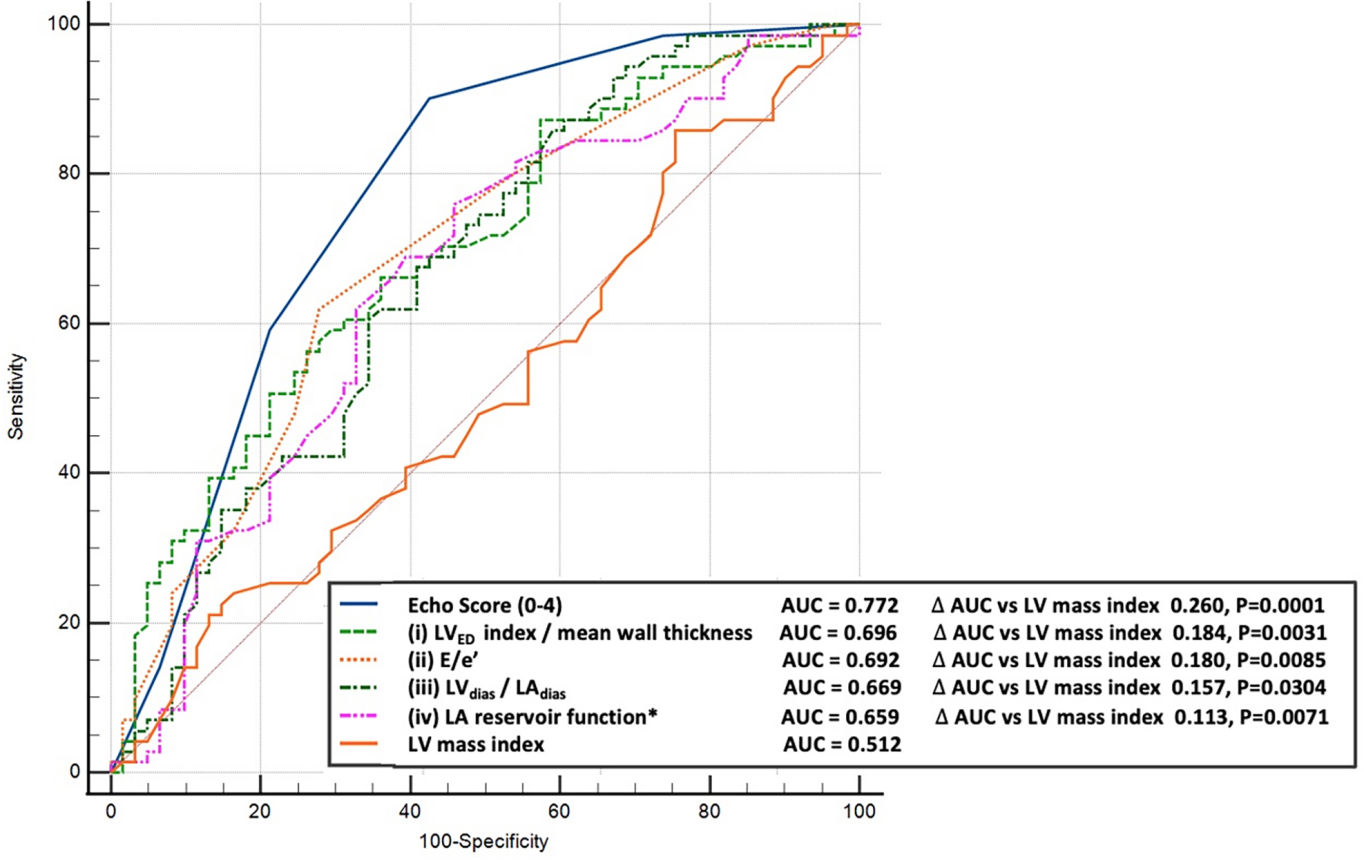
Comparison between the receiver operating characteristic curves for diagnosis of hypertension. Top-echo score (blue line), middle-selected individual echocardiographic parameters (dotted coloured lines) compared to left ventricular mass index (bottom brown line).

**Figure 3:**
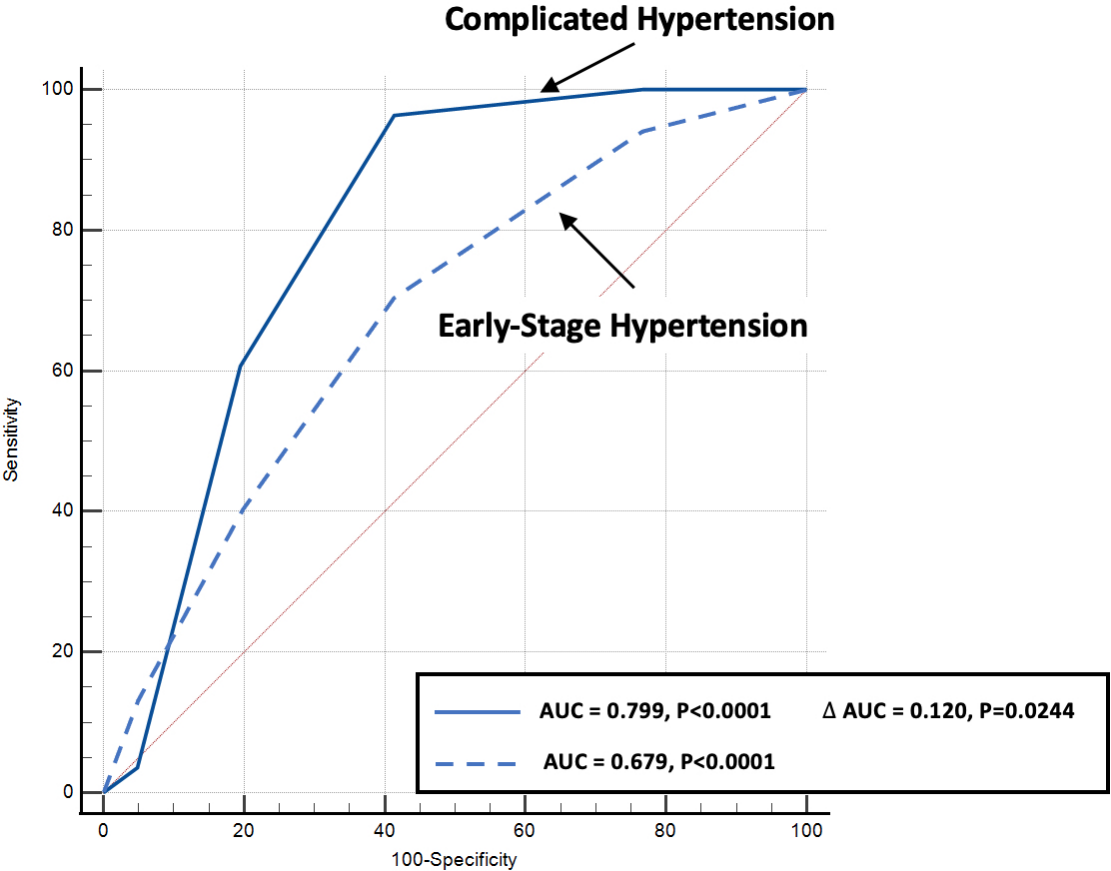
Comparison of echo score receiver operating characteristic curves for the diagnosis of complicated (solid blue line) as compared to early-stage hypertension (dotted blue line) patients.

Predictive modelling for TTE data includes the parameters listed in Table 3. Doppler-derived E/e’>8, LV concentricity/remodelling measured by the ratio of LV_ED_ index/mean wall thickness ≤57, ratio of LV_dias_/LA_dias_ ≤1.62, and LA reservoir function ≤0.58 had a high discriminatory power to diagnose HHD (AUC: 0.690, 0.651, 0.619, and 0.600, *p* < 0.0001, *p* = 0.0003, *p* = 0.0052, and *p* = 0.0194, respectively). These four TTE-derived parameters were chosen for their overall accuracy in predicting HHD. They were grouped to obtain an overall simplified diagnostic TTE (LEDA) score. This method provided the highest discriminatory power with an AUC of 0.772 and overpowered single TTE measurements as well as the LV mass index (Figure 2). The LEDA score was graded accordingly, where 0 was the minimum and 4 was the maximum score obtained if each of the four parameters scored one point. The description of each parameter with their corresponding cut-off values and the probability of underlying HHD based on the LEDA score are shown in Table 4. A LEDA echo score of 0 indicated a 0% probability of the presence of HHD, but a score of 4 indicated a 95% probability of underlying HHD. Logistic analysis confirmed that the proposed echo score allowed for the differentiation of complicated from uncomplicated patients with early-stage hypertension (ΔAUC = 0.120, *p* = 0.0244) (Figure 3). LEDA was positively correlated with the severity of underlying hypertension and patient age (Table 5). Furthermore, LEDA was found to be closely linked to the presence of type 2 diabetes (likelihood ratio: 16, *p* = 0.0036) and Stage 3 chronic kidney disease (likelihood ratio: 12, *p* = 0.0190). A borderline but not significant relationship was observed between LEDA and obstructive sleep apnoea (likelihood ratio: 9, *p* = 0.0556), as well as mild to moderate coronary artery disease (likelihood ratio: 9, *p* = 0.0525). LEDA score did not exhibit a significant association with smoking history (likelihood ratio: 2, *p* = 0.7895) or underlying hyperlipidaemia (likelihood ratio: 7, *p* = 0.1423).

**Table 4:** Echo score (LEDA) of left heart remodelling indices characteristics for hypertensive heart disease. Top – transthoracic echocardiographic parameters with cut off value and one point for each measurement. Bottom – total score with corresponding probability of underlying hypertension.

	Parameter	Cut-off value	Points
**L**	**L**eft ventricle end-diastolic volume index/mean left ventricle wall thickness (LV_ED_ index/Mean wall thickness, ratio)	≤57	1
**E**	**E**arly diastolic mitral inflow to mitral annuls velocity (E/e’, ratio)	>8	1
**D**	Left ventricle **D**iastasis volume to left atrial diastasis volume (LV_dias_/LA_dias,_ ratio)	≤1.62	1
**A**	Left **A**trial reservoir function ((LA_max_-LA_min_)/LA_max_)	≤0.58	1
** **	**LEDA** echo score		Sum (0–4)

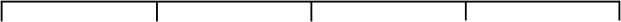
 Total Points 0 1 2 3 4

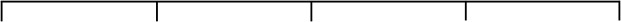
 Probability of hypertensive heart disease 0 23 59 80 95%

**Table 5:** Correlation between the LEDA^a^ score and clinical variables.

Parameter	Correlation coefficient	95% confidence interval lower	95% confidence interval upper	*p*-value
Age (years)	0.453	0.328	0.563	<0.0001
Blood pressure grade (number)	0.331	0.193	0.455	<0.0001
Blood pressure medications (number)	0.314	0.176	0.441	<0.0001
Office blood pressure systolic, mmHg	0.143	−0.004	0.284	0.0571
Office blood pressure diastolic, mmHg	0.056	−0.091	0.202	0.4542
Body surface area, m^2^	−0.031	−0.177	0.117	0.6821
Body mass index	0.054	−0.094	0.199	0.4769
Heart rate, beats/min	−0.028	−0.174	0.120	0.7105
Depression Anxiety Score (20)	−0.007	−0.195	0.181	0.9420
Quality of life (EuroQol 5 Dimension 5 Level) score (21)	0.103	−0.086	0.286	0.2843

^a^ **LEDA** score of four parameters (0–4): (i) **L**eft ventricular end-diastolic volume index/mean left ventricular wall thickness ≤57, (ii) **E**arly diastolic mitral inflow/mitral annulus velocity >8, (iii) left ventricular **D**iastasis volume/left atrial diastasis volume ≤1.62, and (iv) left **A**trial reservoir function ≤0.58.

## Discussion

The cumulative effects of hypertension on cardiac remodelling and vascular aging are well recognised (22). Therefore, the detection and monitoring of HHD are important in clinical practice, with the overarching goal of improving diagnosis rates and long-term prognoses.

Hypertension is commonly associated with an increase in LV wall thickness, leading to LV hypertrophy. This phenomenon signifies the maladaptive response to chronic pressure overload, eventually resulting in contractile dysfunction (10–14, 23). LV hypertrophy and LV mass are widely used measures for assessing established features of HHD and are familiar to clinicians (24). Understanding the sequence of adaptive changes in left heart anatomy and function, which ultimately culminate in alterations in LV geometry, filling pressure, and the development of LV hypertrophy, remains a topic of interest (25, 26).

In this study, we prospectively analysed changes in left heart remodelling in patients with hypertension, excluding those with underlying myocardial ischaemia or obstructive coronary disease. We used TTE-derived parameters, including LV concentricity, filling pressure, and LA function. In addition, we expanded our observations by introducing a parameter obtained from both TTE and CTCA, specifically measured during diastasis: the left heart chamber volume ratio (LV_dias_/LA_dias_).

### LA volume and function

Larger LA volumes were noted in diastasis (LA_dias_) and end-diastole (LA_min_), but not in systole at LA_max_. This pattern of LA dilatation in mid to late diastole, rather than in late systole, has been documented previously, and is associated with increasing LV filling pressure, consequent elevation in LA pressure, and, finally, augmented LA wall stress (26, 27). Simultaneously, we observed a reduction in LA reservoir function. These findings support the notion that the reduction in LA reservoir function occurs at an earlier stage, while LA systolic dysfunction (pump function) emerges later in the progression of HHD, often coinciding with HF symptoms. This systolic dysfunction is often secondary to LV structural remodelling and LA afterload mismatch (28, 29). LA diastolic filling, specifically reservoir filling, depends on both LA relaxation and compliance, with the latter being a determinant of stroke volume and an important determinant of LA function (29). A recent study has demonstrated that reduced LA reservoir function is associated with increased mortality in Patients with HF (30).

### Volume ratio analysis

The value of the ratio of LA to LV diameter, as a single TTE non-invasive marker of LV compliance, has been investigated previously and has been observed to be ≥1.0 in patients with hypertension and diabetes (31). In addition, the ratio of LA to LV end-diastolic volume, measured using magnetic resonance imaging, has been shown to be a strong predictor of HF incidence, atrial fibrillation, and coronary disease mortality (32), while CTCA volumetric data obtained from retrospective acquisition was applied to evaluate diastolic dysfunction and to predict HF (33).

In the current study, we incorporated our previous concept (19) by analysing the ratio of LV to LA volume measured in diastasis, obtained from both TTE and CTCA examinations. Notably, even in the absence of LV dilatation, LV hypertrophy defined by either LV mass index or LV mass/height^2.7^, and with no changes in LA_max_, we observed a reduction in the LV_dias_/LA_dias_ ratio in patients with hypertension. Power analysis revealed that both TTE- and CTCA-derived LV_dias_/LA_dias_ ratios accurately distinguished patients with HHD, with cut-off values of ≤1.62 and ≤1.76, respectively.

The potential advantage of LV_dias_/LA_dias_ is that it is a simple and easy to obtain parameter available from most standard TTEs and, as examined in this study, is valuable when using both TTE and CTCA. Whether this method is applicable to other cardiac imaging techniques remains to be determined.

### Combined TTE scoring index

We introduced a novel TTE-derived score, the LEDA score, comprising four parameters to aid in the identification of HHD: (1) LV_ED_/index/mean wall thickness ≤57, (2) E/e’ ratio by Doppler >8, (3) LV_dias_/LA_dias_ ≤1.62, and (4) LA reservoir function ≤0.58. The concept of combining these four parameters surpassed the diagnostic utility of individual LA or LV TTE parameter, as well as the LV mass index (Figure 2). Each parameter, if present, contributes one point to the score, allowing for a maximum total score of four for an individual patient. A score of four yielded a 95% probability of the presence of HHD. LEDA was positively correlated with the severity of underlying hypertension and patient age, and was also found to be closely linked to the presence of type 2 diabetes and Stage 3 chronic kidney disease. There was only a borderline correlation with obstructive sleep apnoea and mild to moderate coronary artery disease. There was no correlation between LEDA and body mass index.

## Conclusions

This study offers critical insights into the complex dynamics of left heart remodelling in hypertensive patients. By identifying novel parameters, LV_dias_/LA_dias,_ and introducing the LEDA score, we aim to improve the diagnostic accuracy of hypertension-related cardiac changes. These findings hold promise for enhancing clinical practice and ultimately improving the long-term prognosis of patients with hypertension. More data are needed to validate the LEDA score and age-related changes. One of the potential advantages of LV_dias_/LA_dias_ lies in its simplicity and ease of measurement. It is accessible through most standard TTE examinations, making it readily available in routine clinical practice. Furthermore, our study demonstrated its value when obtained through both TTE and CTCA assessments, suggesting its versatility in different imaging modalities.

## Data Availability

The raw data supporting the conclusions of this article will be made available by the authors, without undue reservation.
